# Endothelial Autophagy: an Effective Target for Radiation-induced Cerebral Capillary Damage

**DOI:** 10.1038/s41598-019-57234-9

**Published:** 2020-01-17

**Authors:** Xiaolin Ai, Zengpanpan Ye, Yuqin Yao, Jianghong Xiao, Chao You, Jianguo Xu, Xi Huang, Jian Zhong, Min Fan, Xuejiao Song, Huashan Shi, Dongmei Zhang, Chengjian Zhao

**Affiliations:** 1 State Key Laboratory of Biotherapy and Cancer Center, West China Hospital, Sichuan University, and Collaborative Innovation Center for Biotherapy, Chengdu, Sichuan P. R. China; 2 Department of Neurosurgery, West China Hospital, Sichuan University, Chengdu, Sichuan P. R. China; 3 West China School of Public Health, No.4 West China Teaching Hospital, Sichuan University, Chengdu, Sichuan P. R. China; 4 State Key Laboratory of Biotherapy and Department of Head and Neck Oncology, West China Hospital, West China Medical School, Sichuan University, Chengdu, Sichuan P. R. China; 5 Department of Imaging, West China Hospital of Sichuan University, Chengdu, Sichuan P. R. China; 6 Department of Gynecology and Obstetrics, State Key Laboratory of Biotherapy, West China Second University Hospital, Sichuan University and Collaborative Innovation Center for Biotherapy, Chengdu, China

**Keywords:** Radiotherapy, Neuro-vascular interactions

## Abstract

Toxicity to central nervous system tissues is the common side effects for radiotherapy of brain tumor. The radiation toxicity has been thought to be related to the damage of cerebral endothelium. However, because of lacking a suitable high-resolution *vivo* model, cellular response of cerebral capillaries to radiation remained unclear. Here, we present the *flk:eGFP* transgenic zebrafish larvae as a feasible model to study the radiation toxicity to cerebral capillary. We showed that, in living zebrafish larvae, radiation could induce acute cerebral capillary shrinkage and blood-flow obstruction, resulting brain hypoxia and glycolysis retardant. Although *in vivo* neuron damage was also observed after the radiation exposure, further investigation found that they didn’t response to the same dosage of radiation *in vitro*, indicating that radiation induced neuron damage was a secondary-effect of cerebral vascular function damage. In addition, transgenic labeling and qPCR results showed that the radiation-induced acute cerebral endothelial damage was correlated with intensive endothelial autophagy. Different autophagy inhibitors could significantly alleviate the radiation-induced cerebral capillary damage and prolong the survival of zebrafish larvae. Therefore, we showed that radiation could directly damage cerebral capillary, resulting to blood flow deficiency and neuron death, which suggested endothelial autophagy as a potential target for radiation-induced brain toxicity.

## Introduction

Most of patients with primary brain tumors or metastases tumors had to undergo radiotherapy, such as whole-brain radiotherapy (WBRT), stereotactic radio surgery (SRS) and intensity modulated radiation therapy (IMRT)^1,2^. Although radiotherapy was an effective treatment for brain tumors^3–5^, experimental and clinical evidence suggested that more than 40% of people receiving radiotherapy would suffer from brain damage and mainly manifested as cognitive dysfunction^6^. Radiation-induced cognitive dysfunction refers to the dysfunction of memory, learning, processing speed, and attention, occurring in 50–90% of these patients and gradually becoming irreversible^2,7,8^. Although radiotherapy-induced brain damage has been discussed in many studies, the cellular and molecular mechanisms of radiation-induced brain damage is still controversial. Radiation can damage neuronal, glial and vascular compartments of the brain and may lead to molecular, cellular and functional changes^9^.

The neurovascular unit (NVU), composed of neurons, interneurons, astrocytes, endothelial cells, basal lamina, and extracellular matrix, is the basal structure of brain homeostasis^10^. these cells work together intimately and be closely coordinated, which ensure dynamic linkages in reciprocal way under physiological conditions^11^. Although the radiotherapy would induce the damage of all components, the endothelial cells were more sensitive to radiation^12,13^ and showed significant increased caspase-3/7 activity in a radiation dose-dependent manner, whereas microglia and neuronal cells exhibited lower ratio of dead cells at each dose studied^14^. As the brain is a constant metabolic demand organ^15^ (consuming 20% of the body), endothelium damage and decreased microcirculation might lead to poor perfusion of brain tissue, which could cause death of other cells (including neurons and glial cells) within the neurovascular unit.

Varies animal models have been applied to study the radiation induced vascular damage, they found that radiation therapy could induce dose-dependent endothelial apoptosis^16^, disruption of the blood-brain barrier^17^, vascular rarefaction, and cognitive deficits^18^. However, few can reveal the *in vivo* morphological and functional changes of cerebral capillaries after exposing to ionizing radiation. The zebrafish has been served as an important animal model to study the geneses of the vascular and nervous system disease^19,20^. The small size, optical translucency, and conservative gene of the zebrafish larvae, in combination with a variety of fluorescent labeling transgenic lines permit real-time and high-resolution observation of morphological and gene-expression changes in endothelial cells *in vivo*.

Therefore, we established a zebrafish model using the larvae of transgenic zebrafish (*flk:eGFP*) to study radiation-induced cerebrovascular damage. Continuously confocal tracking astonishingly discovered the morphologically and functionally changes in cerebral capillaries after radiation. 3D coculture of zebrafish neurons and endothelial cells suggested that endothelial cells were more sensitive to radiation compared with other cells in brain. Whole-brain living images of *fli1a:mCherry-GFP-LC3* transgenic zebrafish larvae and q-PCR data indicated the association of endothelial autophagy with the radiation induced damage to cerebral capillary. Finally, autophagy inhibition experiments highlighted the use of autophagy inhibitors in relieving the damage of cerebral capillaries and the subsequent effect to the neurons and glia after the radiotherapy.

## Materials and Methods

### Clinical research

From the database of West China Hospital, a patient who was diagnosed with glioma and underwent Computed Tomography angiography (CTA) before and after brain radiation was enrolled. The interval time of the patient from first to last radiation was within 40 days, the times of radiation was 10, and total dosage of radiotherapy was 40 Gy. The clinical research has received approval from the institutional review board of the Medical Faculty at the West China Hospital, Sichuan University and all methods were carried out in accordance with relevant guidelines and regulations. The written informed consent was obtained from this patient.

### Transgenic zebrafish maintenance and establishment

Lines used in this study included *flk:eGFP* (zfin: s843Tg), *HuC:mCherry* (zfin: nia02Tg). *GFAP:mCherry* and *fli1a:mCherry-GFP-LC3* were established using multisite gateway system (Life Technologies) and Tol2 kit^21^. Zebrafish were maintained at 28.5 °C with a 14-hour light/10- hour dark cycle as previously described^22^. Embryos were kept in incubator at 28.5 °C, and treated with 0.1 Mm 1-phenyl-2-thiourea (PTU, Sigma P5272) to inhibit pigment formation beyond 24 hours post-fertilization. All zebrafish experiments were conducted in accordance with the guidelines of the Animal Care and Use Committee of Sichuan University (Chengdu, Sichuan, China) and approved by the institutional review board of the Medical Faculty at the West China Hospital, Sichuan University.

### 3-D culture of transgenic zebrafish neurons and endothelial cells

*flk:eGFP* and *HuC:mCherry* transgenic zebrafish were incrossed and the embryos were collected. At 80% epiboly/tailbud stage, embryos were washed with PBS containing 5X antibiotics. All embryos were then dechorionated at six hours post fertilization using pronase enzyme degradation. Twenty embryos in each group were then dissociated into single cells with continuous pipetting. Zebrafish embryos cells were then seeded into matrigel in 48-well plates for 1 week. The cells were cultured by complete DMEM/F12 medium (Gibco) supplemented with epidermal growth factor (EGF, 20 ng/ml, Peprotech), basic fibroblast growth factor (bFGF, 10 ng/ml, Peprotech) and B27 (1 × , Invitrogen). Before or after the ionizing radiation, the cells were generally maintained at 28 °C with 5% CO_2_.

### X-ray radiation

Radiation experiments were performed according to the Radiation Safety Manual of West China Hospital, Sichuan University. Briefly, zebrafish larvae (8dpf, days post fertilization) were stably mounted in the central of 2.5 cm thick 3% Sodium carboxymethlycellulose (SCMC). Larvae mounted in 6-well dish were then directly exposed to X-Ray Radiation (ELEKTA, Versa HD, Sweden) with 10 cm × 10 cm of filed size at West China Hospital. Larvae in the radiated group received a single 5 Gy and 10 Gy dose radiation respectively. Control larvae were treated identically but received no radiation. The larvae were put back into incubator within 30 min after radiation exposure.

### Imaging strategy and quantification

For imaging cerebral vasculature of zebrafish *in vivo*, selected embryos were mounted in low melting point (LMP) agarose (1%, wt/vol) at the bottom of a 29 mm glass bottom dish and covered with fish water at 33 °C. The real-time tracking images were taken with Zeiss LSM 880 Confocal Microscope with Airyscan (Carl Zeiss, Germany). The midbrain region of each brain were imaged with a confocal microscope under 40× lens with a total depth of 100 μm. We counted LC3 puncta on all blood vessels, and calculated the number of puncta on each 100μm vessels. We calculated the number of all perfused vessel branches and vascular diameter basing on fluorescent images by ZEN blue and analyzed by the Prism software.

### Brain angiography and cerebral blood volume monitor

For intracranial angiography of zebrafish larva *in vivo*, fluorescein sodium (Sigma F6337, 376 Da) and Dextran blue (MW, 10 kDa) were dissolved in PBS to final concentrations of 2 mg/ml and 12.5 mg/ml respectively. Zebrafish larvae were anesthetized with 0.2 mg/ml Tricaine (Sigma A5040) before the tracker injection. For brain angiography, about 3 nl of tracers were injected into each larva from pericardium and confocal live images were taken within 1–2 hours post the tracer injection. The whole brain perfusion images were taken with Confocal Microscope. The brain of zebrafish was moved to the center of the field of vision, and all images generally had a same depth (100 μm). We calculated number of all perfused vessel branches in fluorescent images and quantified by the Prism software. For the cerebral blood-volume monitor assay, 1 hour after the tracer injection, the brains of the zebrafish larvae (n = 6 in each group) were dissected out using tweezers under a stereomicroscope. The brains were then completely digested using 100 μl of mixture of 0.25% trypsin, 2.5 mg/ml collagenase IV and 1 mg/ml DNAses. After high-speed centrifuge (2000 rpm/min) for 10 min, the fluorescent intensity of fluorescein sodium and Dextran blue in the supernatants were measured using a fluorescence microplate reader (FLx800™).

### Hypoxia detection assay and NAD(P)H autofluorescence assessment

Intracranial hypoxia was evaluated using a hypoxia-detecting probe (MAR, Goryo Chemical, Japan) as the manufacturer described^23^. Briefly, hypoxia-detecting probe was injected directed into the zebrafish brain at 4-day post radiation (dpr) and the injected zebrafish were then maintained in incubator for 2 hours. Then the zebrafish larvae were fixed with 4% PFA and the brains were isolated with tweezers under a stereomicroscope. After staining, the fluorescence intensity of the zebrafish brain was analyzed by Confocal (Leica SP5). For NAD(P)H assessment, the brain samples were fixed with 4% PFA for 4 hours, and then washed with PBS for 1 hour. NAD(P)H had its own autofluorescence, and it absorbs light of wavelength 340 ± 30 nm and emits fluorescence at 460 ± 50 nm^24^. The specific fluorescent intensity of NAD(P)H in each sample was imaged using a confocal (Leica SP5), and quantified by image J.

### Quantitative real-time PCR

The cerebral endothelial cells of flk:eGFP transgenic zebrafish larvae are showing green fluorescence and the cells were isolated by FACS. Total RNA was extracted using an RNeasy column (Axygen), and cDNA was obtained using SuperScript III reverse transcriptase (Invitrogen). Quantitative PCR (qPCR) was then performed on an ABI7300 thermocycler (Applied Biosystems) using SYBR green Master Mix (Applied Biosystems). The relative fold change of autophagy related genes, including *lc3, beclin1, atg5* and *ambra*, were calculated in 0, 5 and 10 Gy group at 2-day and 4-day post radiation. The following primers were used: *β-actin* (CTTCTTGGGTATGGAATCTTGC and GTACCACCAGACAATACAGTG); *lc3* (GAGAAGTTTTTGCCGCCTCT and ACCTGTGTCCGAACATCTCC); *atg5* (AGGATACCCGCCTGTTTCAC and TCCCTCGTGTTCAAACCACA); *beclin1* (CATCACTGAGAACGAGTGCCA and CTGTGGTTGCGTCCCTCATC); *ambra* (CTGCTGCTCATTGCCACC and CTGCTCCTCATGCTGACC).

### Flow cytometry

To test survival of neurons and glial cells after radiation, the zebrafish brains were dissected and digested on 4day post radiation as previous described. DAPI/AnnexinV-FITC staining was performed according to the standard method^25^. Flow Cytometry data were acquired by FACSVerse (BD Biosciences) and analyzed by FlowJo software (Tree Star). The apoptosis test of neurons and endothelial cells in 3-D culture system was performed using the same DAPI/AnnexinV-FITC staining in a similar way.

### Drug administration details

A clinical anti-vasoconstriction drug nimodipine and three autophagy inhibitors (Wortamanin, Ly294002 and Chloroquine) (Selleck). These small molecular inhibitors were added directly into fish water. Nimodipine (5 μM) was immediately added into fish water after the radiation exposure, and the drug was changed daily for continuing 4 days. Living brain imaging by confocal was done at 2 and 4dpr. For the autophagy inhibitor treat groups, zebrafish embryos were soaked in fish water containing autophagy inhibitors (Wortamanin (1 μM), Ly294002 (10 μM) and Chloroquine (100 μM)) for 6 hours each day, and then washed and changed with fresh water until the death of these radiated larvae.

### Statistical analysis

Prism software was used for statistical analysis. All datasets were challenged by a normality test. Datasets with a Normal distribution were analyzed by unpaired t test. For zebrafish survival studies, Kaplan–Meier survival curves were generated and analyzed for statistical significance with GraphPad Prism. A level of *P < 0.05 or **P < 0.01 was regarded as statistically significant.

## Results

### Whole-Brain-Radiation Therapy impairs the intracranial blood-perfusion in a glioma patient

**Figure 1:**
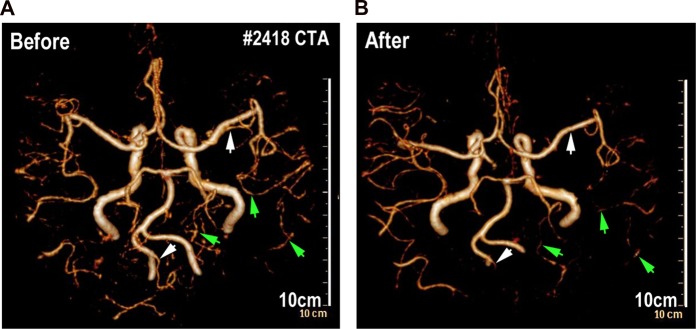
Whole brain radiations decrease the density of cerebral vessels in human patient. (**A**) CTA vascular reconstruction images depicting cerebral vascular of patients before whole-brain radiotherapy (left) and 4 months after whole-brain radiotherapy (right). The arrowheads indicate cerebral vascular before whole-brain radiotherapy (left) and after (right).

A patient from West China Hospital was collected in the present study. This patient was diagnosed with glioma and underwent Computed Tomography angiography (CTA) before and after brain radiation. The patient totally received 10 times of whole brain radiation (the total radiotherapy dosage is 40 Gy) within 40 days from June 2016 to July 2016. After comparing 3-dimensional computed tomography angiography (Fig. 1), we found that the intracranial vessels and blood-perfusion was significantly affected after the last time of radiation. Some big intracranial vessels (such as left post cerebral artery) (Fig. 1) shrank after 10 times of brain radiation and partial small cerebral vessels (such as branches of right middle cerebral artery and branches of post cerebral artery) (Fig. 1) could no longer be detected by CTA after the radiation. This piece of clinical data from this glioma patient implies that intensive brain radiation might result damage of intracranial vessels and reduction of the cerebral blood-perfusion.

### X-ray radiation results acute shrinkage of cerebral capillaries of zebrafish larva

**Figure 2:**
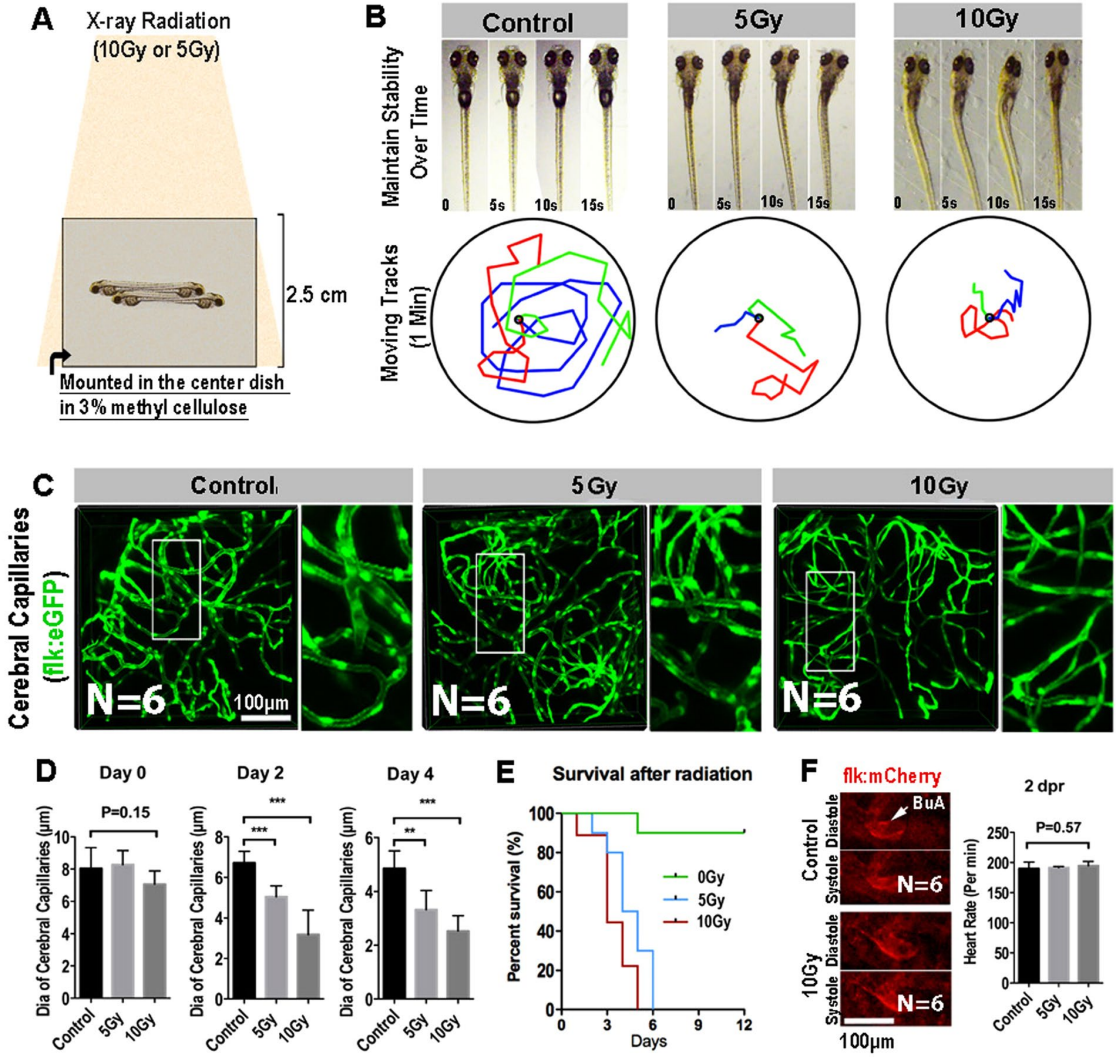
Radiation specifically damages the brain capillaries of transgenic zebrafish. (**A**) A schematic representation of X-ray radiation for transgenic zebrafish at 0, 5 and 10 Gy respectively. (**B**) The ability of maintain stability over 15 s (top) and moving tracks of zebrafish over 1 minute (bottom) at 2-day post radiation in 0, 5 and 10 Gy group (n = 3 zebrafishes per group). (**C**) Live example of images (each representative of 6 zebrafishes) showing the midbrain region of the zebrafish larva, and depicting endothelial cells (green) of cerebral capillaries in 0, 5 and 10 Gy group at 4-day post radiation. The right images are magnifications of the boxed areas in left images. Scale bars, 100 µm. (**D**) Morphometric analyses of cerebral capillaries diameter in 0, 5 and 10 Gy group at 0 day, 2-day, 4-day post radiation respectively (n = 6 zebrafishes per group). (**E**) The survival analysis of zebrafishes in 0, 5 and 10 Gy group (n = 10 zebrafishes per group). (**F**) Live example of images (each representative of 6 zebrafishes) depicting systole and diastole of heart (red) in 0 and 10 Gy group at 2-day post radiation (left), and heart rate of zebrafishes per minute in control, 5 and 10 Gy group at 2-day post radiation (right). Statistical analysis in D and F was performed using t-test: **P < 0.05, ***P < 0.01. Data represent the mean ± s.e.m.

Zebrafish has been envisioned as a popular vertebrate model system for studying radioprotection^26^. Here, to establish a zebrafish X-ray Radiation damage model, 8dpf zebrafish embryos were stably mounted in the central of 2.5 cm thick 3% Sodium carboxymethlycellulose (SCMC). Mounted Larvae were exposed to a single dose of X-Ray Radiation (5 Gy and 10 Gy), and then were released to fresh water for viability and behavior assessment (Fig. 2A). At 2dpr, we found, although the zebrafish morphology looks normal, most of the radiated-larvae had obvious balance and coordination problems (Fig. 2B). Larvae couldn’t keep themselves steady in the water and kept readjusting their bodies (Fig. 2B, upper panel). In addition, by track the swim trajectories, we found that the radiated-larvae were reluctant to swim, comparing with the control larvae at the same age (Fig. 2B, lower panel).

Then, to investigate the acute changes of cerebral vasculature of the zebrafish larvae exposing to radiation, we employed the *flk:eGFP* transgenic zebrafish. The endothelial cells of the *flk:eGFP* zebrafish were GFP labeled and the zebrafish larvae were transparent, which enable us to image and reconstruct the whole cerebral vasculature of zebrafish larva at single-cell resolution by confocal. Interestingly, living images of the brain vasculature revealed that the general diameter of cerebral capillaries of the radiated-larvae were smaller than that in the untreated zebrafish (Fig. 2C). Quantitative analysis indicated that the shrinkage of cerebral capillaries was radiation dosage dependent (Fig. 2C,D). In addition, time-series evaluation results indicated that the radiation-induced capillary shrinkage was irreversible over time (Fig. 2D) and most of the radiated-larvae (5 Gy and 10 Gy) would die, within 6 days post the radiation (Fig. 2E).

We also imaged and evaluated the cardiovascular system and the cardiac function of the larvae after exposing to radiation. Fluorescent images indicated that the size and movement of Bulbus Arteriosus (BuA) during a cardiac cycle was similar to the untreated larvae (Fig. 2F) and the heart beat rate was also not affected (p = 0.57, Fig. 2F). These results suggested the relatively normal cardiovascular function of zebrafish larvae after exposing to radiation. Altogether, our results showed that single dose of X-ray radiation could specifically induce an irreversible cerebral capillary damage in zebrafish larvae.

### Radiation-induced cerebral capillary shrinkage affects cerebral blood-perfusion in zebrafish larvae

To monitor the cerebral blood flow in zebrafish larva after exposing to radiation, Dextran blue (10,000 MW) and fluorescein sodium (376 MW) were pericardially injected into *flk:eGFP* zebrafish larva at different time points before the imaging and evaluation.

**Figure 3:**
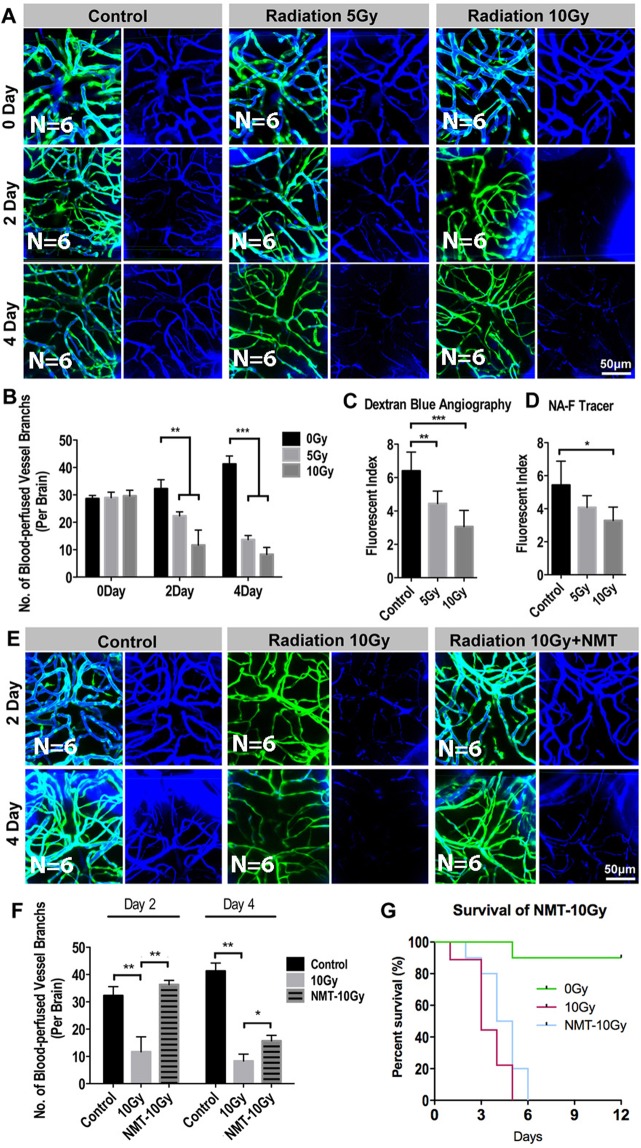
Radiation-induced endothelial damage results insufficient blood perfusion into the brain. (**A**) Live example of images (each representative of 6 zebrafishes) depicting endothelial cells (green) of cerebral capillaries (right) and the tracer Dextran blue (MW 10 kDa) pericardial injected (left) in 0, 5 and 10 Gy group at 0 day, 2-day and 4-day post radiation. Scale bars, 100 µm. (**B**) Morphometric analyses of blood-perfused vessel branches in 0, 5 and 10 Gy group at 0 day, 2-day, 4-day post radiation respectively (n = 6 zebrafishes per group). (**C**,**D**) The fluorescence index of the zebrafishes whole brain injected with Dextran blue (**C**) and fluorescein sodium (Sigma F6337, 376 Da) (**D**) tested by fluorescein microplate reader in 0, 5 and 10 Gy group at 4-day post radiation (n = 6 zebrafishes per group). (**E**) Live example of images (each representative of 6 zebrafishes) depicting blood perfusion of zebrafishes brain in 0, 10 Gy and 10 Gy + Nimodipine group at 2-day and 4-day post radiation. Scale bars, 100 µm. (**F**) Morphometric analyses of blood-perfused vessel branches in 0, 10 Gy and 10 Gy + Nimodipine group at 2-day and 4-day post radiation (n = 6 zebrafishes per group). (**G**) The survival analysis of zebrafishes in 0, 10 Gy and 10 Gy + Nimodipine group (n = 10 zebrafishes per group). Statistical analysis in (**C**,**D**,**F**) was performed using t-test: **P < 0.05, ***P < 0.01. Data represent the mean ± s.e.m.

The whole brain confocal images revealed that the blue-dextran-labeled blood-flow in the cerebral capillaries continuing declining after exposing to the radiation (Fig. 3A,B). In addition, to quantify the changes of blood volume in the zebrafish brain, at different time points (0, 2, 4 dpr), we dissected the larva brains (n = 10 in each group at every time point) under a stereomicroscope. The brains in each group were completely digested and the fluorescence intensity (dextran blue and fluorescein sodium) of the supernatant was normalized and measured (Fluorescence Microplate Reader, Thermo). Consistent with the angiography of cerebral vessels, we found that the fluorescent intensity of the radiated-brains supernatant was much lower than that in the untreated group (Fig. 3C,D), suggested a reduction of the blood volume in the radiated brains.

Brain blood-volume reduction could be resulted from the cerebral capillary shrinkage that induced by radiation. To clarify this relationship, we tried to rescue the brain blood flow defect by using a clinical anti-vasoconstriction drug-Nimodipine. Nimodipine is a calcium channel blocker and has some selectivity for cerebral vasculature. Nimodipine (5 μM) was directly added into fish water after the radiation exposure, and the drug was changed daily for continuing 4days. Living brain imaging by confocal was done at 2 and 4dpr. As expected, with the Nimodipine treatment, obvious vasodilation of the cerebral capillaries of the radiated-larvae was observed at 2dpr and the cerebral blood-perfusion showed a marked improvement (Fig. 3E,F). However, this improvement only lasts for a short time. At 4dpr, even with the continuing Nimodipine treatment, the cerebral blood-perfusion in the radiated-larva was dramatically dropped and severely vascular shrinkage was detected (Fig. 3E,F). As a result, the short-term effect of Nimodipine on the cerebral vasoconstriction only resulted in a limited over-all survival improvement after the radiation (Fig. 3G).

### Radiation-induced cerebral blood-perfusion deficiency results secondary neuron and glial cells damage in zebrafish brain

To investigate the influence of radiation-induced cerebral vascular damage, we carried out a series of *in vivo* and *in vitro* experiments. Firstly, by measuring intensity of the NAD(P)H-related autofluorescence in the zebrafish brain^27^, we monitored the changes of cerebral energy metabolism of zebrafish larvae after exposing to radiation.

**Figure 4:**
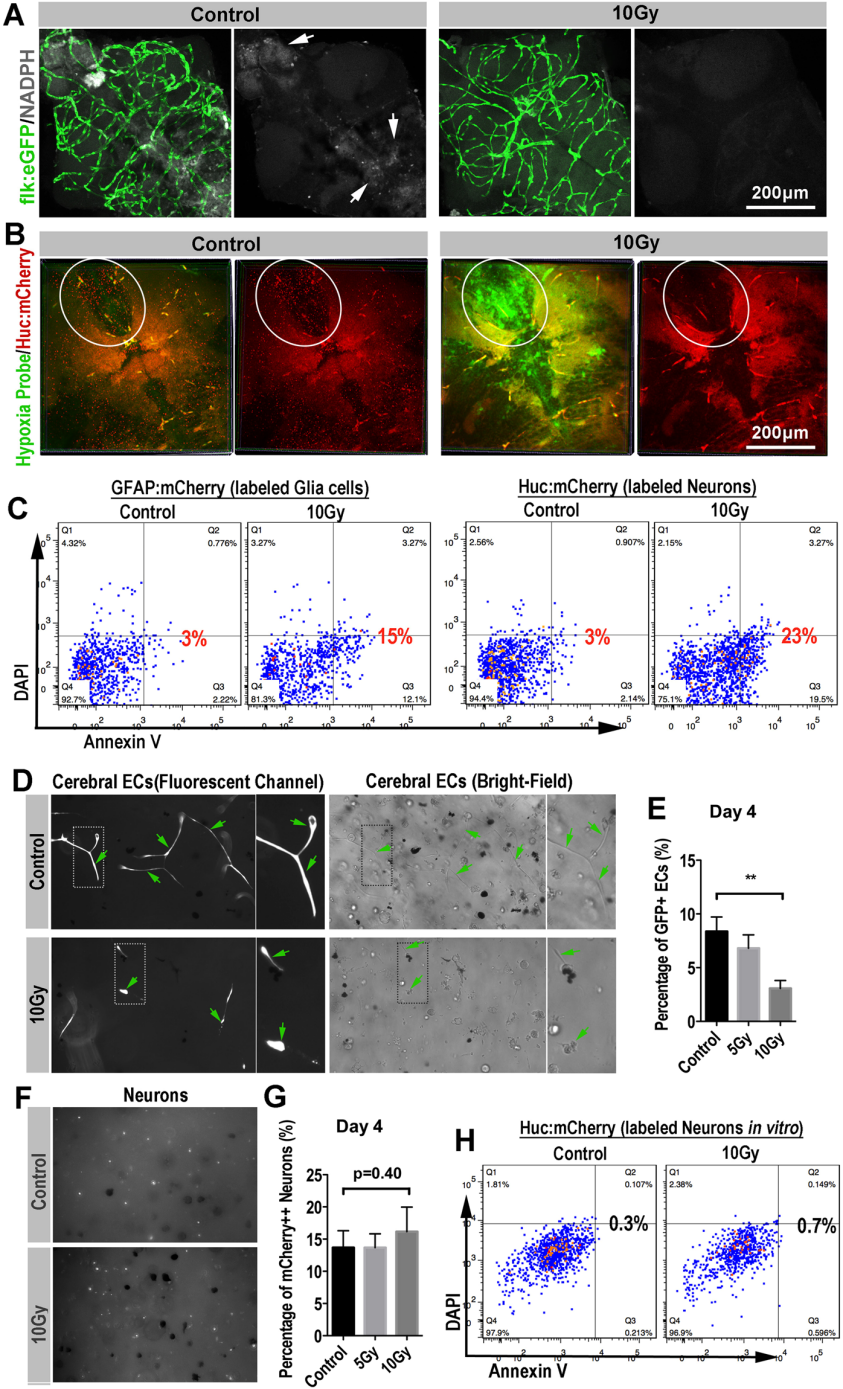
Secondary blood-perfusion insufficiency induced the apoptosis of neuron and glial (*in vivo/in vitro*). (**A**) Example of images (each representative of 6 zebrafishes) depicting endothelial cells (green) of cerebral capillaries (left) and spontaneous fluorescence (white) of NADPH (right) in 0 and 10 Gy group at 4-day post radiation. Scale bars, 100 µm. (**B**) Example of images (each representative of 6 zebrafishes) depicting intracranial injection of hypoxia probe (green) and neurons (red) of *HuC:mCherry* zebrafish in 0 and 10 Gy group at 4-day post radiation. The boxed areas indicated the changes in severity of hypoxia and changes in the number of neurons between 0 and 10 Gy groups. Scale bars, 100 µm. (**C**) *In vivo*, glial cells (*GFAP:mCherry* zebrafish lines) and neurons (*HuC:mCherry* zebrafish lines) were gated for apoptosis staining in control and 10 Gy group at 4-day post radiation (n = 6 zebrafishes per group). (**D**) Example of images (each representative of 6 visions) depicting *vitro* 3-D culture of cerebral endothelial cells in 0 and 10 Gy groups at the fluorescence channel (left) and bright field (right). The arrowheads and boxes indicate the apoptosis of endothelial cells in 10 Gy group compared with 0 Gy group. (**E**) Flow analysis of percentage of endothelial cells *in vitro* 3-D culture at 4-day post radiation among 0, 5 and 10 Gy groups. (**F**) Example of images (each representative of 6 visions) depicting *vitro* 3-D culture of neurons (white) in 0 and 10 Gy groups at 4-day post radiation. (**G**) Flow analysis of percentage of neurons *in vitro* 3-D culture at 4-day post radiation among 0, 5 and 10 Gy groups. (**H**) Neurons (*HuC:mCherry* zebrafish lines) *in vitro* 3-D culture were gated for apoptosis staining in 0 and 10 Gy group at 4-day post radiation (n = 6 zebrafishes per group). Statistical analysis in (**E**,**G**) was performed using t-test: **P < 0.05, ***P < 0.01. Data represent the mean ± s.e.m.

The NADPH auto fluorescent signaling is mainly located in the telencephalon, diencephalon and mesencephalon of zebrafish brain (Fig. 4), which is generally overlapped with the regions with high neuron metabolism. In accordance with the shrinkage of the cerebral capillaries, Confocal images showed that the NAD(P)H-related fluorescence in the radiated-brains was dramatically reduced at 4dpr (Figs. 4A, S1). Next, by using the hypoxia detection probe together with the neuron-labeled transgenic zebrafish (*HuC:mCherry*), we revealed the severe hypoxia (green channel) in the zebrafish brains at 4dpr (Fig. 4B). At the same time, we found that rare healthy neurons (mCherry^+^) were detected in the radiated-brains at 4dpr, compared with the untreated zebrafish brains (Fig. 4B).

Vertebrate neurons are generally viewed as among the most anoxia-sensitive of all cells^28,29^. The death of neurons from hypoxia and glucose deprivation in the radiated brains can follow quickly from necrosis or in other slower ways, such as apoptosis. To determine the possible role of programmed cell death in the radiated zebrafish brains, we then applied annexin V apoptosis assay. To monitor the neuron and glial cells in the zebrafish brain, we used either neurons-labeled *HuC:mCherry* or the glia-labeled *GFAP:mCherry* transgenic zebrafish in the radiation test. At 2dpr, we digested the brains of the transgenic zebrafish larvae (n = 10 in each group) that with or without radiation exposure (10 Gy) and staining of DAPI/FITC-annexin V was then applied.

Flow cytometry results indicated that higher apoptosis ratio of neurons and glial cells in the brains with radiation exposure.

To confirm that the neuron damage was mainly a secondary effect of radiation-induced cerebral blood flow deficiency, rather than a direct toxicity of radiation. We co-cultured the zebrafish neurons and endothelial cells in 3D Matrigel and exposed these cells to X-ray radiation at the same dose as we tested in zebrafish (5 and 10 Gy). The co-cultured neurons and endothelial cells were maintained at 28 °C with 5% CO2, supplied with sufficient glucose and essential growth factors (methods and materials). At 4dpr, we evaluated the status of endothelial cells (*flk:eGFP*) and neurons (*Huc:mCherry*). As showed in the fluorescent channel (Fig. 4D), control endothelial cells in the 3D Matrigel behaved as normal, forming endothelial sprouts and connecting with each other. However, the radiated endothelial sprouts were much thinner and were more retracted than the control (Fig. 4D). Endothelial sprouts in the Matrigel were then digested into single cells and counted. The result indicated that, except for the morphological changes, there were actually less endothelial cells survived in the wells after the radiation and the cell number reduction was radiation-dose dependent (Fig. 4E). On the other hand, we also evaluated the cell status of the neurons in the 3D Marigel after the radiation as we did with endothelial sprouts. Interestingly, contrasts to the endothelial cells, neurons were more resistant to radiation. No detectable morphological or cell number changes were observed (Fig. 4F,G). In addition, annexin V apoptosis assay indicated that no significant apoptosis was initiated in the culturing neurons after exposing to radiation (Fig. 4H).

These results together indicated that endothelial cells, compared with neurons, are more sensitive to radiation. And it’s highly possible that the *in vivo* neuron death after the radiation was attributed to the radiation-induced cerebral damage and intracranial ischemia.

### Radiation induces intensive autophagy flux in the cerebral endothelial cells in zebrafish larvae

**Figure 5:**
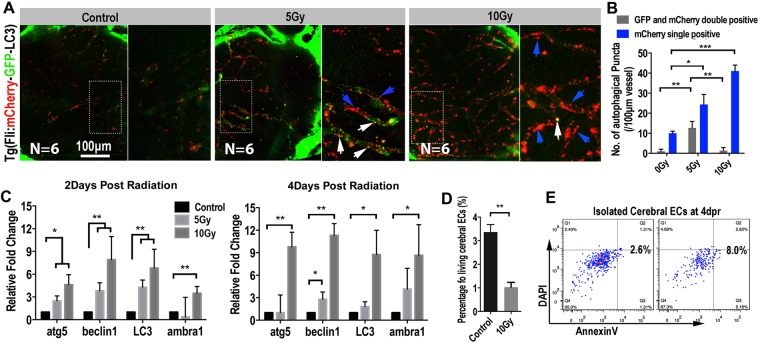
Extensive endothelial autophagy was induced by the radiation in the brain. (**A**) Example of images (each representative of 6 zebrafishes) depicting autophagosome (green) of endothelial cells and autolysosome (red) of endothelial cells in 0 and 10 Gy group at 4-day post radiation. The right images are magnifications of the boxed areas in left images. Scale bars, 100 µm. (**B**) Morphometric analyses of GFP and mCherry double positive puncta (yellow puncta) and mCherry single positive puncta in 0, 5 and 10 Gy group at 4-day post radiation. The right images are magnifications of the boxed areas in left images. Scale bars, 100 µm. (**C**) Real-time quantification of single-cell mRNA expression levels of autophagy-related genes in radiation induced zebrafish endothelial cells at 2-day, 4-day post radiation respectively (n = 3 endothelial cells per group). Gene expression levels were normalized to those of β-actin in the control group. (**D**) Endothelial cells (*flk:eGFP* zebrafish lines) *in vitro* 3-D culture were gated for apoptosis staining in 0 and 10 Gy group at 4-day post radiation (n = 6 zebrafishes per group). Statistical analysis in (**C**) was performed using t-test: **P < 0.05, ***P < 0.01. Data represent the mean ± s.e.m.

The radiation-induced endothelial damage results in severe cerebral capillary shrinkage, which leads to intracranial ischemia and the death of neurons and glial cells. However, the mechanism of radiation induced endothelial damage remains unclear. Previous studies showed that radiation almost uniformly promotes autophagy in tumor cells^30^ and extensive autophagy has been related to programmed cell death^31^. Thus, to track the possible endothelial autophagy flux after radiation exposure, we established a transgenic zebrafish line (fli:mCherry-GFP-LC3), in which the endothelial cells specifically expressing chimeric LC3 fused to both GFP (acid sensitive) and mCherry (acid stable). This chimeric LC3 protein has been used to monitor autophagosome transport^32,33^. The fli:mCherry-GFP-LC3 transgenic zebrafish larvae (8dpf) were exposed to X-ray radiation at different dosages as we did before. Confocal images at 4dpr showed that, contrasts to the untreated larvae, abundant LC3 puncta were detected in the cerebral endothelium of radiated zebrafish larvae (Fig. 5A). Quantitative analysis indicated that the number of the LC3 puncta were radiation dosage dependent (Fig. 5B). In addition, in the 5 Gy group, partial of the puncta were GFP and mCherry double positive, indicating that these autophagosomes had not yet fused with the lysosomes. And in the 10 Gy group, most of the LC3 puncta were mCherry single positive, indicating the maturation of these autophagosomes (Fig. 5A,B).

To confirm the activation of endothelial autophagy after the X-ray radiation, we then isolated cerebral endothelial cells by FACS from zebrafish brains with or without radiation exposure. The mRNA expression levels of autophagy related genes (*lc3, beclin1, atg5* and *ambra1*) were then evaluated by qPCR. At both 2 and 4 dpr, the qPCR results showed that the expression of evaluated autophagy-related genes were elevated in the cerebral endothelial cells of radiated brains, compared with that in the control larvae.

Previous studies have correlated the intensive autophagic flux with various forms of cell death^34^. Here, in our experiment, we found that the cerebral endothelial cells in the radiated zebrafish brains also showed a higher expression of apoptotic genes (Fig. S2). Then, using AnnexinV/DAPI apoptosis assay, we indeed found a higher apoptotic ratio and lower percentage of living endothelial cells in the radiated zebrafish brains were than that in the control brains (Fig. 5D,E). Hence, these results together suggested a role of radiation-induced endothelial autophagic damage or autophagic death in the radiation-induced cerebral vascular damage and intracranial ischemia.

### Inhibition of autophagy alleviates radiation-induced cerebral vascular damage and extends the survival of zebrafish larvae

Autophagy is a double-edged sword, either keeping the cells survived through adverse microenvironment or directly incorporating in various forms of cell death. Here, we revealed the general cerebral endothelium damage after exposing to radiation at different dosages (5 and 10 Gy), we rationalized that radiation-induced endothelial autophagy was positively correlated to the cerebral capillary damage. To test this hypothesis, we tried several autophagy inhibitors: Wortamanin, Ly294002 and Cloroquine. PI3K is required for autophagy^35^ and inhibition of PI3K with LY294002 or Wortamanin can inhibit autophagic sequestration. Chloroquine inhibits autophagy as it raises the lysosomal pH, which leads to inhibition of both fusion of autophagosome with lysosome and lysosomal protein degradation^36^.

**Figure 6:**
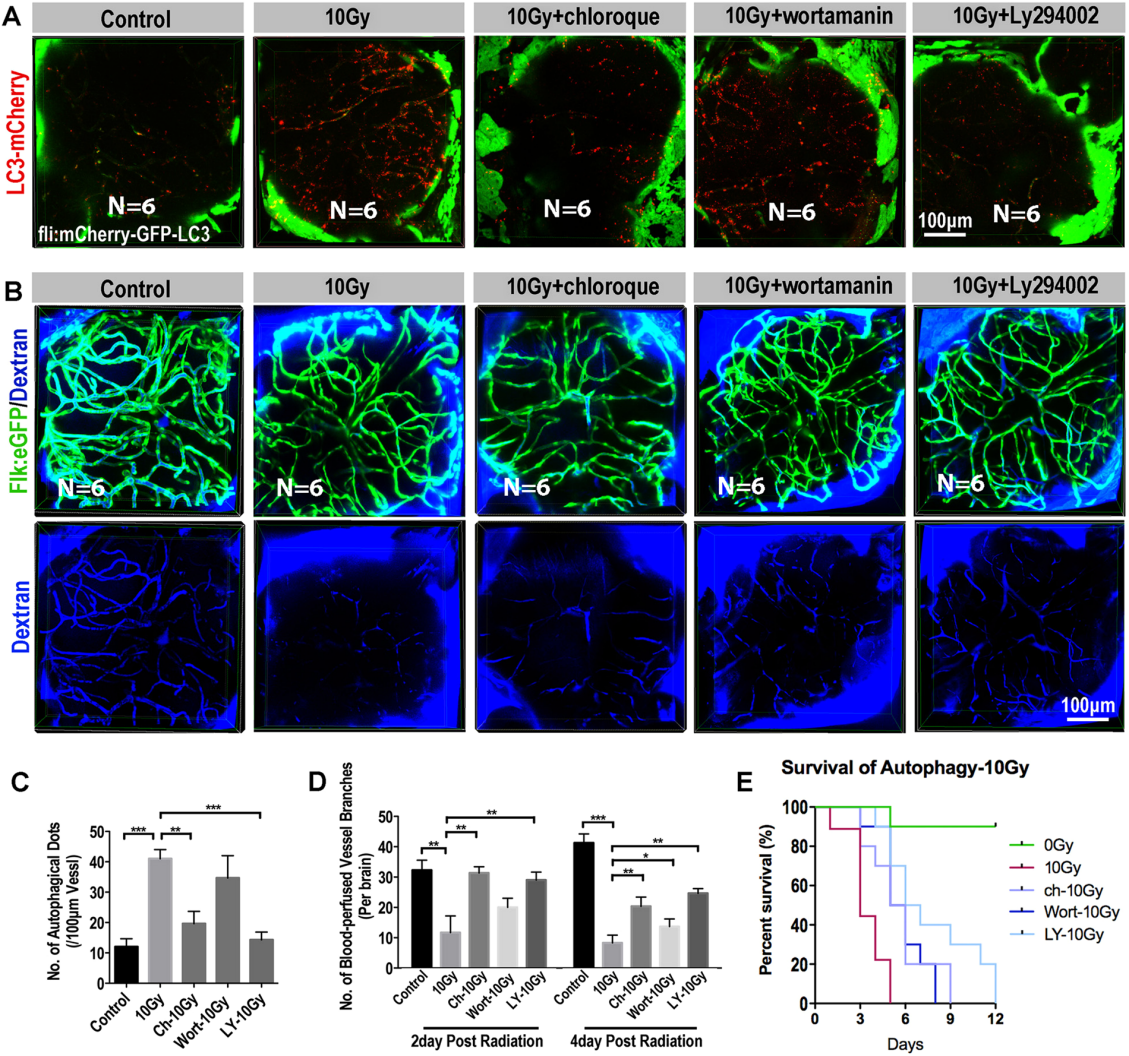
Inhibition of autophagy enhanced the blood perfusion into the brain b (**A**) Example of images (each representative of 6 zebrafishes) depicting autophagosome (green) of endothelial cells and autolysosome (red) of endothelial cells in 0, 10 Gy group, 10 Gy + chloroque group, 10 Gy + wortamanin group and 10 Gy + Ly294002 group at 4-day post radiation. Scale bars, 100 µm. (**B**) Live example of images (each representative of 6 zebrafishes) depicting endothelial cells (green) of cerebral capillaries (top) and the tracer Dextran blue (MW 10 kDa) pericardial injected (bottom) in 0, 10 Gy group, 10 Gy + chloroque group, 10 Gy + wortamanin group and 10 Gy + Ly294002 group at 4-day post radiation. Scale bars, 100 µm. (**C**) Morphometric analyses of autophagical puncta in 0, 10 Gy group, 10 Gy + chloroque group, 10 Gy + wortamanin group and 10 Gy + Ly294002 group at 4-day post radiation (n = 6 zebrafishes per group). (**D**) Morphometric analyses of blood-perfused vessel branches in 0, 10 Gy group, 10 Gy + chloroque group, 10 Gy + wortamanin group and 10 Gy + Ly294002 group at 2-day and 4-day post radiation (n = 6 zebrafishes per group). (**E**) The survival analysis of zebrafishes in 0, 10 Gy group, 10 Gy + Cloroquine (n = 10, p = 0.0126), 10 Gy + ly294002 (n = 10, p < 0.01) and 10 Gy + Wortamanin (n = 10, p < 0.01). Statistical analysis in C and D was performed using t-test: **P < 0.05, ***P < 0.01. Data represent the mean ± s.e.m.

After exposing to radiation, zebrafish larvae were soaked in culturing water supplied with Wortamanin (1 μM), Ly294002 (10 μM) or Cloroquine (100 μM) for 6 hours per day until the death of these radiated larvae. The dosages of anti-vasoconstriction drug Nimodipine and autophagy inhibitors Wortamanin, Ly294002 and Cloroquine were determined by zebrafish maximum tolerated concentration and maximum patient’s plasma concentration of each compound. At 4dpr, Confocal images of *fli:mCherry-GFP-LC3* transgenic zebrafish larvae showed that that number of fluorescent LC3 puncta in the cerebral endothelium were significantly reduced with the treatment of autophagy inhibitors (Fig. 6A,C) after radiation exposure. In addition, by using the *flk:eGFP* transgenic larvae and the Dextran-blue angiography, we evaluated the changes of cerebral capillaries and blood flow. As expected, with the treatment of autophagy inhibitors, the blood-perfusion in the brains of radiated larvae was significantly improved (Fig. 6B,D) and the survival of radiated zebrafish larvae were also significantly extended, especially for the group that treated with ly294002 (Fig. 6E, p < 0.01). To study the change of zebrafish behavior caused by radiation after using autophagy inhibitors, we evaluate their stability and moving tracks. We found the control radiated-larvae had obvious balance and coordination problems, and the radiated zebrafish larvae using autophagy inhibitors showed more active and could keep balance (Fig. S3). These results indicate that radiation-induced intensive endothelial autophagy is positively correlated to the cerebral vascular damage and could be served as a target for anti-radiation-induced brain toxicity.

## Discussion

Previous studies have suggested that endothelial cells were more sensitive to radiation compared with neurons and glial cells; therefore, brain injury induced by radiotherapy may be related to vascular damage^12^. To test brain vascular pathogenicity *in vivo*, we developed a transgenic zebrafish radiation model to study the radiation-induced cerebrovascular damage, which allowed real-time living tracking of endothelial damage and blood perfusion in brain followed by ionizing radiation. In particular, the *(fli:mCherry-GFP-LC3)* transgenic zebrafish and the qPCR analysis indicated that cerebral endothelial autophagy was significantly associated with the radiation-induced cerebral capillary damage. Further autophagy inhibition experiments demonstrated a vital role of autophagy in radiation-induced endothelial damage, highlighting the therapeutic potential of regulating autophagy for the treatment of radiation-induced brain injury.

Using *flk:eGFP* fish, we demonstrated the death of cerebral vascular endothelial cells induced by radiation appeared in a dose-dependent pattern. Recent studies suggested radiation induced cerebral vascular damage begins with progressive endothelial cells loss. In a rat radiation model, single dose of 5–20 Gy radiation in brain lead to a 15% decrease of endothelial cell number within 1 day, then followed by continuous endothelial cells loss within a month^37^. In addition, we observed contraction of cerebral capillaries induced by radiation, and radiation could further result in cerebral capillaries network simplification over time. Initial studies demonstrated that fractionated radiotherapy induced a rarefaction of vessels within the Cornu Ammonis 1(CA1), Cornu Ammonis 3(CA3) and dentate gyrus region of the hippocampus (an area important for spatial memory), which is closely related to supply of oxygen, nutrients and trophic factors to brain, and thus result in deficits in spatial learning and memory^38^. However, in some mouse radiation damage models, the blood perfusion of tissue cannot be accurately assessed, and the damage of vascular function cannot be evaluated at the whole brain level. In addition, the tissue fixation and section staining would alter the vascular structure. Meanwhile, because of the relatively low resolution of CTA and MRA for human patients, morphological and functional changes of micro vessels after radiation could not be detected either. In our study, the whole brain of transgenic zebrafish can be directly imaged under a fluorescent microscope or confocal due to their small size and transparency. Therefore, using zebrafish radiation model, the 3-D *in vivo* tracking of brain vasculature provides chances for us to preciously evaluate the cerebral endothelium and blood perfusion after exposing to ionize radiation.

The molecular mechanism of cerebral vascular damage by ionizing radiation is still controversial. Radiation could induce the damage of DNA followed by increased expression of apoptosis signals in many vascular endothelial cells, resulting in angiogenesis compromise^14^, which is partially consistent with our results. The DNA damage response coordinates many processes, including DNA repair, regulation of cell cycle checkpoints, and ultimately induction of a programmed cell death, most often apoptosis, when DNA damage cannot be repaired. Many researchers suggest, that autophagy, a catabolic process considered to be a cellular survival mechanism, is a central player in the regulation of DNA damage response. For mild stress, activation of DNA damage response may evoke autophagy as an early adaptive response. For serious stress, such as radiotherapy, autophagy did not promote survival, but induced apoptosis.

We found that the apoptosis of endothelial cells increased to 8% after radiation compared with control group (2%) in zebrafish brain. Besides, the strongly upregulation of autophagy genes^39^, including atg5, beclin-1, LC3 and ambra1, demonstrate that the endothelial autophagy is activated by ionizing radiation. To lively image the radiated-induced endothelial autophagy in zebrafish, we establish the fli:mCherry-GFP-LC3 transgenic zebrafish to monitor the autophagy process specifically in endothelial cells after X-ray radiation. Interestingly, our results demonstrated that radiation could lead to acceleration of autophagic flux and apoptosis in cerebral endothelial cells (Fig. 5). However, in an *in vitro* study, Kalamida *et al*. found ionizing radiation could trigger perinuclear accumulation of the autophagosomal proteins and repression of the autophagolysosomal flux in human umbilical vein endothelial cells, suggesting against the blockage of autophagic flux may contribute to radio-resistance^40,41^. The difference might owe to the difference between *in vivo* and *in vitro* studies, and different radiation dosage and animal models.

Autophagy is a highly conserved cytoprotective and complex process, and several pharmacological and nutritional interventions are available to inhibit autophagy at the nucleation, elongation, fusion or degradation phase. Although chloroquine, ly294002 and wortamannin are all considered as autophagy inhibitors, each functions differently in the autophagy process. Chloroquine^42^ is a inhibitor of lysosomal function, ly294002^43^ is VPS34 inhibitor, and they can pass through blood-brain barrier. Compared with ly294002, wortamannin^44^ had no blood-brain barrier permeant and had relatively bad selectivity. Although chloroquine and ly294002 are able to significantly repress the formation of autophagosomes in the cerebral endothelium, they had systemically toxic to zebrafish. Commercial autophagy drug library contains hundreds of autophagy related compounds. Take advantage of the high throughput drug screening ability of zebrafish model, we may find an autophagy inhibitor that could significantly alleviate the radiation-induced cerebral vascular damage but has relatively low toxicity in the future.

Our study also had some limitations. For instance, in our current study, the major molecular mechanism of radiation induced vascular damage was tested in zebrafish model, which should be further tested in mammals and human cerebral endothelial cells. In addition, because of the tiny size of zebrafish, the same dose of radiation in zebrafish might be different in patients, which need to further addressed in the following studies. Despite these limitations, it was the first time that revealing the radiation-induced morphological change of cerebral vessels at the whole-brain level. We also tested a mechanism of radiation-induced endothelial damage in transgenic zebrafish model, suggesting a novel therapeutic strategy for radiation-induced cerebral vascular damage.

## Supplementary information

Supplementary Information.
