# Supplementary material for: Endothelial Autophagy: an Effective Target for Radiation-induced Cerebral Capillary Damage
Source: Sci Rep. 2020 Jan 17;10:614. doi: 10.1038/s41598-019-57234-9 (PMC6968992; doi:10.1038/s41598-019-57234-9)
Supplement: Supplementary file 1 — Supplementary Information. [file 41598_2019_57234_MOESM1_ESM.docx]

**Supplementary files: Endothelial Autophagy: an Effective Target for Radiation-induced Cerebral Capillary Damage**

Xiaolin Ai ^1,2#^, Zengpanpan Ye ^1,2#^, Yuqin Yao^3^, Jianghong Xiao^4^, Chao You^2^, Jianguo Xu^2^, Xi Huang^1^, Jian Zhong^1^, Min Fan^5^, Xuejiao Song^3^, Dongmei Zhang^6^, Huashan Shi^1,3*^, Chengjian Zhao^1*^

1. State Key Laboratory of Biotherapy and Cancer Center, West China Hospital, Sichuan University, and Collaborative Innovation Center for Biotherapy, Chengdu, Sichuan, P. R. China.
2. Department of Neurosurgery, West China Hospital, Sichuan University, Chengdu, Sichuan, P. R. China.
3. West China School of Public Health, No.4 West China Teaching Hospital, Sichuan University, Chengdu, Sichuan, P. R. China.
4. State Key Laboratory of Biotherapy and Department of Head and Neck Oncology, West China Hospital, West China Medical School, Sichuan University, Chengdu, Sichuan, P. R. China.
5. Department of Imaging, West China Hospital of Sichuan University, Chengdu, Sichuan, P. R. China.
6. Department of Gynecology and Obstetrics, State Key Laboratory of Biotherapy, West China Second University Hospital, Sichuan University and Collaborative Innovation Center for Biotherapy, Chengdu, China.

Running title: endothelial autophagy: radiation induced CNS damage

Corresponding author: Chenjian Zhao; Mailing address: State Key Laboratory of Biotherapy and Cancer Center, No.17, 3rd, Renmin South Road, Chengdu, Sichuan, China; Phone Number: +86 18328342430; Email address: [chjianzhao@scu.edu.cn](mailto:chjianzhao@scu.edu.cn).

^#^ YZ and AX contributed equally to this work.

*Corresponding author: Huashan Shi and Chengjian Zhao

This work was supported by grants from the National Natural Science Foundation of China (Grant no. 81703064; 81773097; 81872068; 81872391).

Conflicts of interest: No conflict of interest.

**Supplementary file contains: 1 supplementary figure and supplementary experimental procedure.**

**Supplementary Figures:**

**Figure S1
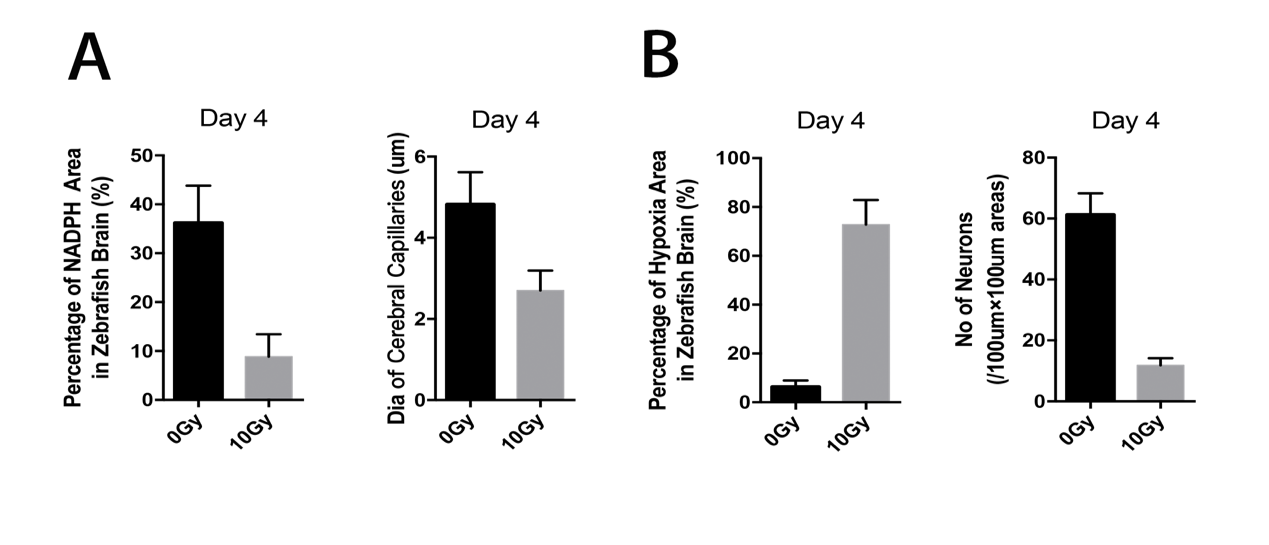
**

(A) Immunofluorescence quantification for percentage of NADPH area (white) in zebrafish brain and diameter of cerebral capillaries (green) and in 0 and 10Gy group at 4 days post rdiation, p<0.01. (B) Immunofluorescence quantification for percentage of hypoxia area (green) in zebrafish brain and number of neurons (red) of 100um ×100 um areas in 0 and 10Gy group at 4-day post radiation, p<0.01.

**Figure S2**

**
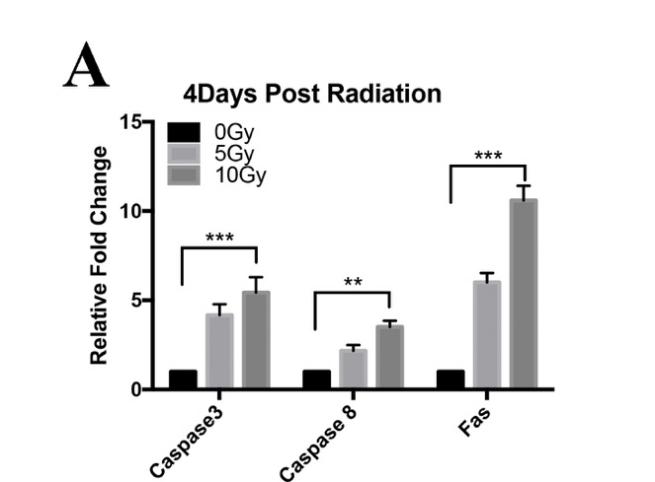
**

(A) Real-time quantification of mRNA expression levels of apoptosis-related genes in radiation induced zebrafish endothelial cells at 2 days, 4 days post radiation respectively (n=3 endothelial cells per group). Gene expression levels were normalized to those of [β](http://www.baidu.com/link?url=fDwggIz39AWcyGm4PkkNRoHEynpy_pIAxG8rSpfbCV-Gbibho37FYaONgWA10NDNLWLrhMtSrUC7AZmuOhp9FAICXjnWsFbuNpLiKZBqwHC)-actin in the control group.

**Figure S3**

**
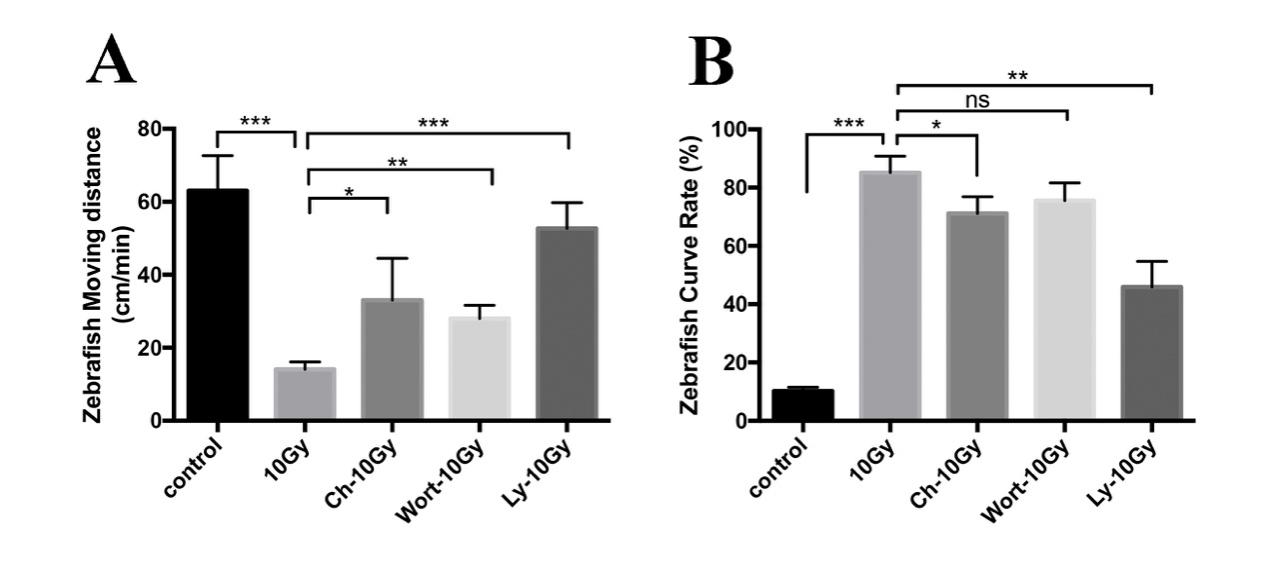
**

(A) The ability of moving distance over 1 minute at 4-day post radiation in 0 and 10Gy group after using autophagy inhibitors (n=3 zebrafishes per group). (B) The zebrafish curve rate at 4days post radiation in 0 and 10Gy group after using autophagy inhibitors (n=3 zebrafishes per group).
